# The low IGFBP-3 level is associated with esophageal cancer patients: a meta-analysis

**DOI:** 10.1186/s12957-016-1055-6

**Published:** 2016-12-15

**Authors:** Guiqin Song, Kang Liu, Xiaoyan Zhu, Xiaolin Yang, Yuewu Shen, Wan Wang, Guidong Shi, Qing Li, Yi Duan, Yunxia Zhao, Gang Feng

**Affiliations:** 1 Department of Biology, North Sichuan Medical College, Nanchong, 637000 Sichuan Province People’s Republic of China; 2 Institute of Tissue Engineering and Stem Cells, North Sichuan Medical College, Nanchong, 637000 Sichuan Province People’s Republic of China; 3 Biotherapy Center, Nanchong Central Hospital, Nanchong, 637000 Sichuan Province People’s Republic of China; 4 Department of Parasitology, North Sichuan Medical College, Nanchong, 637000 Sichuan Province People’s Republic of China; 5 Department of Chest Surgery, The Affiliated Hospital of North Sichuan Medical College, Nanchong, 637000 Sichuan Province People’s Republic of China; 6 The clinic medicine of North Sichuan Medical College, Nanchong, 637000 Sichuan Province People’s Republic of China; 7 State Key Laboratory of Biotherapy, Sichuan University, Chengdu, 610041 Sichuan Province People’s Republic of China

**Keywords:** Esophageal cancer, Overall survival, Clinical pathological features, Meta-analysis

## Abstract

**Background:**

Esophageal cancer was a vital cause of cancer-related mortality worldwide, and the insulin-like growth factor-binding proteins (IGFBPs) has been proved to be an important factor of multiple types of tumors. There is a controversy that whether the IGFBP-3 level is associated with the clinical pathological characteristics and overall survival of esophageal cancer patients. Herein, we aimed to comprehensively assess the association between the low IGFBP-3 level and the risk, overall survival and clinical pathological characteristics of esophageal cancer.

**Method:**

We conducted a meta-analysis using seven eligible studies. The overall odds ratios (OR)/relative risk (RR) and their corresponding 95% confidence interval (CI) were calculated for each parameter.

**Results:**

For the risk of esophageal cancer, the OR was 2.342 (*p* = 0.000), indicating that individuals with lower IGFBP-3 level were more likely to suffer from esophageal cancer, compared to those with relatively high IGFBP-3 level. With respect to the 3-year survival rate, the RR was 2.163 (*p* = 0.027), which demonstrated that esophageal cancer patients with low IGFBP-3 level had significantly lower 3-year survival rate; in terms of clinical pathological characteristics, significantly lower IGFBP-3 level was found for patients in all categories; for survival status, patients in low IGFBP-3 level are more likely to be in the dead survival status (OR = 4.480, *p* = 0.000).

**Conclusion:**

Our meta-analysis suggests that for esophageal cancer, the low IGFBP-3 level is associated with high cancer risk, poor prognosis, and unfavorable tumor stage and metastasis.

## Background

Esophageal cancer is listed as the sixth most common cause of cancer-related mortality worldwide, with an estimated 456000 new cases annually in the world [1, 2]. Several factors have been reported associated with esophageal cancer risk such as drinking high-temperature beverages, poor nutritional status, and insufficient intake of vegetables and fruit [3–7]. Due to the asymptomatic nature and the lack of effective approaches for early detection, individuals with esophageal cancer are usually diagnosed with relatively advanced stage, which contributes to limited treatment methods available and poor prognosis [1, 8]. In spite of the new progress in surgery and chemo-radiotherapy for esophageal cancer, the 5-year survival rate is still lower than 30% [9, 10].

The insulin-like growth factor-binding proteins (IGFBPs), together with interacting ligands and receptors, comprise the insulin-like growth factor (IGF) system [11]. IGFBP-3, which is a multifunctional protein, is secreted by many cell types [12]. As a predominantly secreted protein, IGFBP-3 plays important roles in several molecular mechanisms and signaling pathways that regulate cell survival or apoptosis, particularly in the case of tumor [13]. It has been documented that the epigenetic alteration of IGFBP-3 can impact on multiple types of tumors including non-small-cell lung cancer, hepatocellular carcinoma, ovarian cancer, skin cancer, urological cancers, breast cancer, and gastric cancer [14].

As for esophageal cancer, a previous study, aimed to explore the clinical and prognostic significance of IGFBP-3 in patients with esophageal cancer, suggested that both the clinical pathological classifications and poor overall survival were associated with the low IGFBP-3 level [15]. Whereas, study from Sohda et al. demonstrated that no correlation was observed between the serum IGFBP-3 level and clinical pathological features and overall survival for esophageal cancer patients [16]. Herein, we conducted a meta-analysis to comprehensively evaluate the association between the low IGFBP-3 level and the risk, overall survival and clinical pathological characteristics of esophageal cancer.

## Methods

### Search strategy

We conducted a computer-aided literature search of multiple databases including PubMed, EMBASE, Web of science, Springerlink, and ProQuest. The search strategy was the combination of Medical Subject Headings (MeSH) and items such as *esophageal cancer*, *carcinoma of esophagus*, *esophageal carcinoma*, *esophagus cancer*, *oesophageal carcinoma*, and *insulin-like growth factor binding protein-3*, *IGFBP-3*, *IGFBP-III* , *IBP3*, *BP-53*, *IBP-3*, *IGF-binding protein 3*, *IGFBP-3*, *acid stable subunit of the 140 K IGF complex*, *binding protein 29*, *binding protein 53*, *growth hormone-dependent binding protein*. The last retrieval date was October 1, 2016. We also scanned relevant reviews and reference lists of primary identified literatures to avoid any omission of eligible articles.

### Inclusion and exclusion criteria

In order to get reliable estimations, the following strict inclusion criteria were defined in advance: (1) patients in case group were diagnosed as esophageal cancer; (2) studies focused on the expression level of IGFBP-3 between people with and without esophageal cancer; (3) studies regarding to the correlation of IGFBP-3 with clinical characteristics and the survival rate of patients with esophageal cancer, regardless of the sample size and follow-up period; (4) the IGFBP-3 expression level was evaluated by immunohistochemical staining (IHC), enzyme-linked immunosorbent assay (ELISA), flow cytometry (FCM), or reverse transcription-polymerase chain reaction (RT-PCR) approaches. The exclusion criteria were as follows: (1) duplicated studies; (2) studies in which the relevant information was unavailable; (3) some article types such as reviews, meetings, letters, personal communications, comments, and abstracts.

### Data extraction

Two independent reviewers conducted the assessment of eligible literatures based on the above inclusion and exclusion criteria. From the enrolled studies, the following information was collected: first author, year of publication, country of origin, number of participants in case/control group, treatment before study, detection approaches for IGFBP-3 level, median age, median follow-up period, cut-off value of IGFBP-3 level, positivity rate, the relationship between the expression level of IGFPB-3, and the risk and prognosis of esophageal cancer; approaches of adjuvant therapy, and prognostic analysis.

### Statistical analysis

In our study, the clinical pathological characteristics include the age and gender of patients, the tumor location and size, the tumor category (*T*, *N*, *M*, and TNM categories defined as the previous relevant studies [15, 17–19]), and survival status (dead or alive). The overall survival is measured by the 3-year survival rate. If the information for overall survival is in the form of figure, we used Engauge Digitizer version 4.1 (free software down-loaded from http://sourceforge.net) to read the Kaplan-Meier curves for extracting relevant data to calculate the 3-year survival rate. The STATA 12 software (STATA Corp LP, College Station, Texas, United States) was adopted to constructed forest plots, which are graphical forms to exhibit the relative strength of effects in multiple quantitative studies that deliver the same question. We estimated the heterogeneity across included studies using the *I* ^2^ index. If the heterogeneity was small (*I* ^2^ < 50%), the Mantel–Haenszel fixed-effects model was used to calculate the OR or RR with 95% CI. Otherwise, we applied the DerSimonian and Laird (D-L) random-effects model for the calculation of the OR or RR with 95% CI. We used Begg’s funnel plot to illustrate whether there was any indication of publication bias, and when the funnel plot was asymmetric, significant publication bias was considered. The Egger’s test was also introduced to examine the publication bias and *p* < 0.05 was regarded as significant publication bias.

For cancer risk, the OR > 1 signifies that the low IGFBP-3 level is associated with esophageal cancer, and individuals with low IGFBP-3 level are at high-risk of esophageal cancer. In terms of the 3-year survival rate, the RR > 1 indicates that for patients with esophageal cancer, the low IGFBP-3 level is associated with low 3-year survival rate. With respect to the clinical pathological features, the OR > 1 suggests that for patients with esophageal cancer, the low IGFBP-3 level is correlated with the age (patients over 60 years old are more likely to have low IGFBP-3 level), gender (female patients are more likely to have low IGFBP-3 level), tumor location (patients with thoracic tumor location are more likely to have low IGFBP-3 level), tumor size (patients with tumor size less than 6 mm are more likely to have low IGFBP-3 level), tumor category (patients whose tumor cells have regional lymph nodes in *N* category, metastasize to distant organs in *M* category, and whose tumors have extended invasion in other organs in *T* category and are in III–IVstage of TNM category are more likely to have low IGFBP-3 level), and survival status (patients whose survival status are dead are more likely to have low IGFBP-3 level) of patients. The significance level was set to 0.05.

## Results

### Study characteristics

**Fig. 1:**
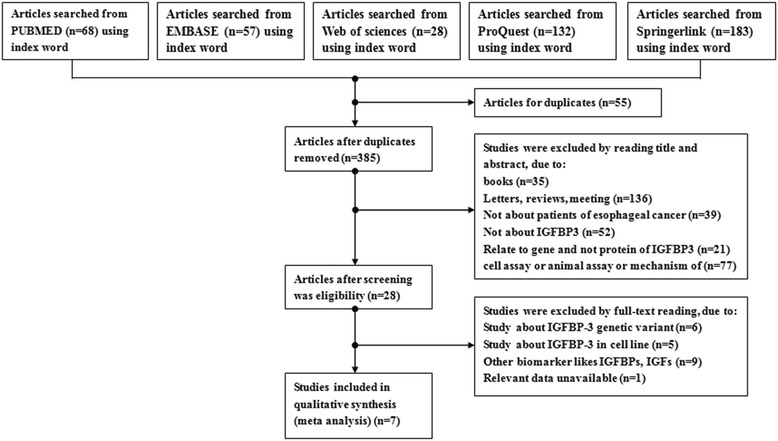
Flow chart of study selection and specific reasons for exclusion from the mete-analysis

With the search strategy, we firstly retrieved 68 articles from PUBMED, 57 from EMBASE, 28 from Web of science, 132 from ProQuest, and 183 from Springerlink. We then removed duplications, leaving 385 articles for further assessment. After reading titles and abstracts, 357 articles were eliminated. Eventually, a total of 7 articles [15–17, 20–23] were eligible for our meta-analysis according to our inclusion and exclusion criteria. Figure 1 showed the flow diagram of study selection and elimination progress. All included articles were published from 2004 to 2016, and their characteristics were displayed in Table 1.

**Table 1:** Characteristics of studies included in the meta-analysis

Study	Population	Case/control (n)	Treatment before study^a^	Test approaches of IGFBP-3 level	Cut-off value	Positivity rate	^b^Results 1	Adjuvant therapy	Prognosis	^c^Results 2
Makoto Sohda (2004) [16]	Japan	66/18	No	ELISA	–	–	High	Surgery	Sur. Survival	No
O. Yilmaz (2006) [21]	Turkey	40/40	No	ELISA	–	–	Low	–	–	–
E. Di Martino (2006) [20]	UK	39/38	No	IHC/RT-PCR	0	76.92%	High	Surgery	–	–
Lei Zhao (2012) [15]	China	110/56	–	IHC	4	51.82%	Low	–	Sur. Survival	High
M. Natsuizaka (2014) [22]	Japan	91/91	–	IHC	0	35.16%	Low	Surgery	Sur. Survival	low
Li-Ling Luo (2015) [32]	China	70/10	Radiotherapy	IHC	0.65	45.71%	High	Radiotherapy	Sur. Survival	High
Peng Ye (2016) [23]	China	264/132	–	IHC	–	–	Low	–	–	–

– not related in the article

^a^ *No* had not undergone surgical intervention, chemotherapy, or radiotherapy

^b^ *Results 1* the relationship between the expression level of IGFPB-3 and esophageal cancer. *High* high expression level of IGFPB-3 was found to be associated with a risk of EC. *Low* low expression level of IGFPB-3 was found to be associated with a risk of EC

^c^ *Results 2*: the relationship between IGFBP-3 and prognosis. *High* a high expression level of IGFPB-3 was found to be associated with improved survival rate of EC. *Low* a low expression level of IGFPB-3 was found to be associated with improved survival rate of EC. *No* no significant association was observed between the IGFBP-3 level and prognosis

### Evaluation of the association between the low IGFBP-3 level and the risk of esophageal cancer

**Fig. 2:**
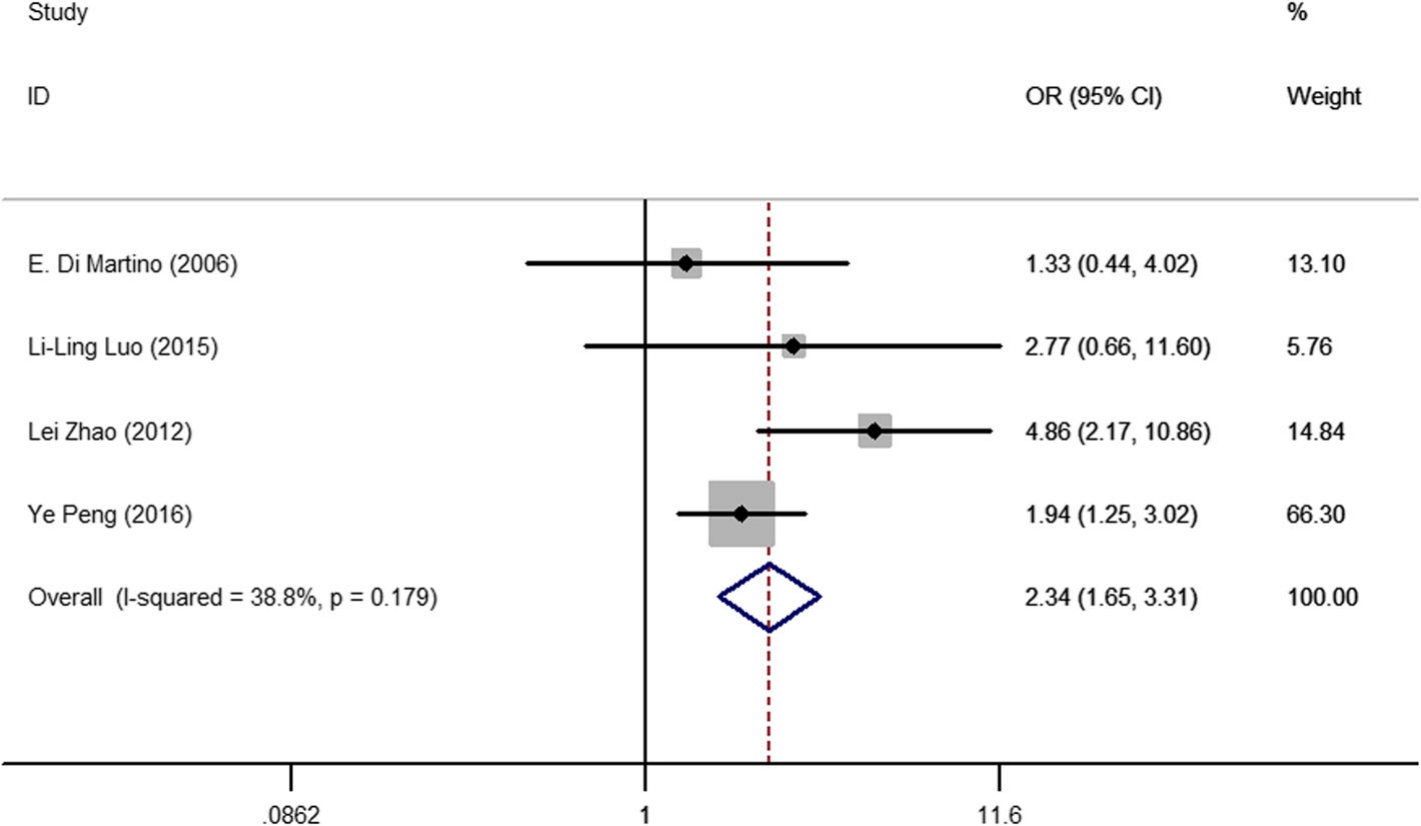
Forest plot of study evaluating the association between the low IGFBP-3 level and the risk of esophageal cancer

There were four eligible studies for the analysis of the correlation between the low IGFBP-3 level and the risk of esophageal cancer. The results were shown in Table 2. The fixed-effects model was used for the calculation of OR and 95% CI, since no significant heterogeneity existed (*I* ^2^ = 38.8%). The OR was 2.342 (95% CI: 1.655–3.313, Fig. 2), and significant difference (*p* = 0.000) was observed in the rate of participants with low IGFBP-3 level between patients with lung cancer and healthy subjects, which suggested the low IGFBP-3 level was associated with high risk of esophageal cancer, and individuals with low IGFBP-3 level were at high risk of esophageal cancer.

**Table 2:** Meta-analysis of IGFBP-3 expressions for esophageal cancer

Groups	*I* ^2^	*P* ^a^ (heterogeneity)	OR	Lower limit	Upper limit	*P* ^b^ (OR)	Begg’s test	Egger’s test
Cancer risk	38.8%	0.179	2.342	1.655	3.313	0.000	0.734	0.434
3-year survival rate	71.20%	0.015	2.163*	1.092	4.287	0.027	0.734	0.814
Age	77.40%	0.035	0.980	0.520	1.860	0.733	1.000	–
Gender	4.70%	0.306	1.250	0.630	2.480	0.548	1.000	–
Location	0.00%	0.962	1.145	0.568	2.307	0.705	1.000	–
Tumor size	72.40%	0.057	1.650	0.900	3.010	0.536	1.000	–
*T* category	19.60%	0.265	2.870	1.510	5.450	0.005	1.000	–
*N* category	0.00%	0.863	3.211	1.561	6.607	0.002	1.000	–
*M* category	0.00%	0.380	3.270	1.670	6.400	0.001	1.000	–
TNM stage	0.00%	0.621	4.110	1.850	9.160	0.000	1.000	–
Survival status	0.00%	0.861	4.490	2.207	9.093	0.000	1.000	–

* value of RR

– unavailable

^a^The *P* value for heterogeneity

^b^The *P* value for OR

### Evaluation of the association between the low IGFBP-3 level and the 3-year survival rate of esophageal cancer

**Fig. 3:**
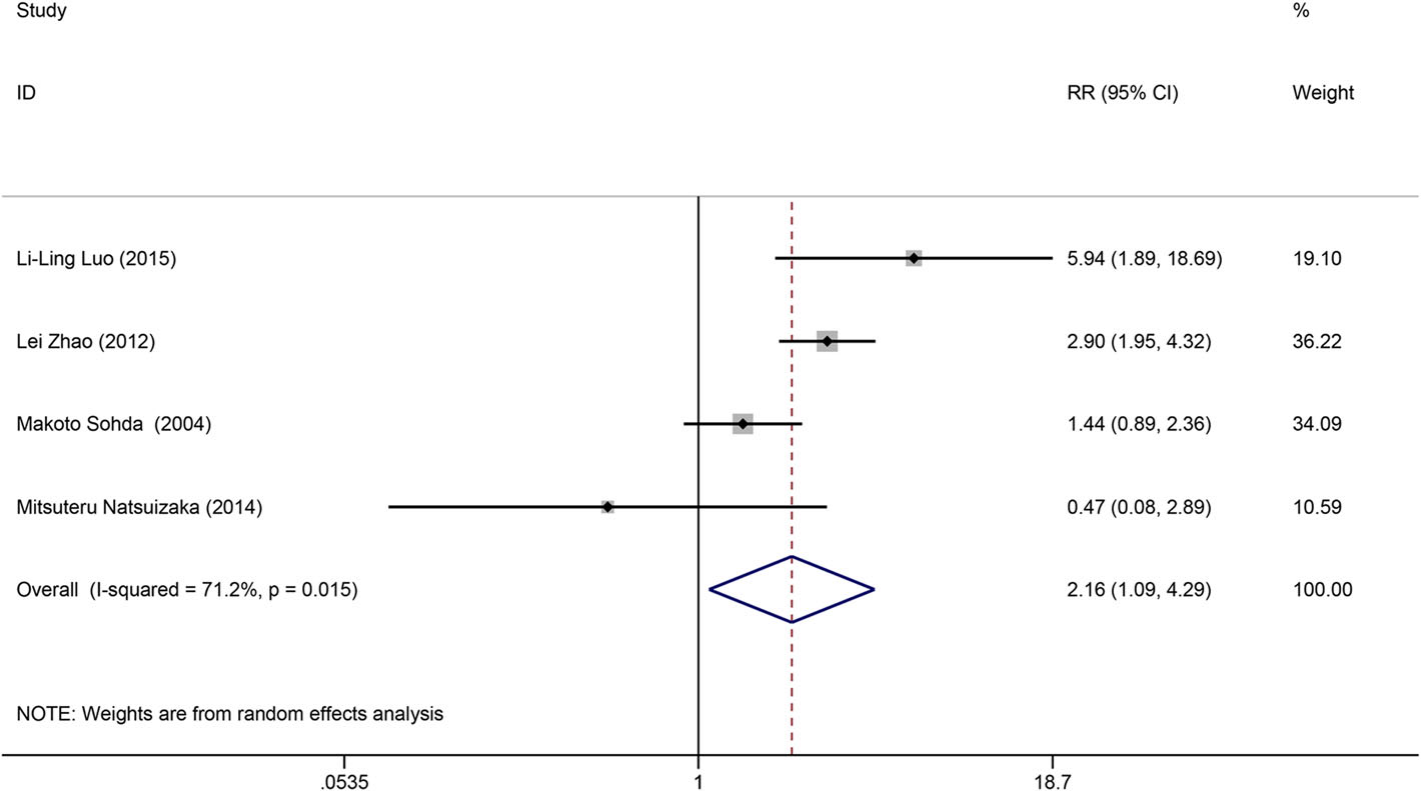
Forest plot of study assessing the association between the low IGFBP-3 level and the 3-year survival rate of esophageal cancer

As for the overall survival with the 3-year survival rate as the endpoint, four studies were enrolled into the analysis and the results were displayed in Table 2. Considering the large heterogeneity (*I* ^2^ = 71.2%), the random-effects model was selected to calculate the RR and its corresponding 95% CI. The RR (RR = 2.163, 95% CI: 1.092–4.287, Fig. 3) was higher than one and the *p* was lower than 0.05 (*p* = 0.027), indicating that the low IGFBP-3 level was associated with low 3-year survival rate, and for esophageal cancer patients, the 3-year survival rate of patients with low IGFBP-3 level was lower than that in patients with relatively high IGFBP-3 level.

### Evaluation of the association between the low IGFBP-3 level and clinical pathological characteristics of esophageal cancer

**Fig. 4:**
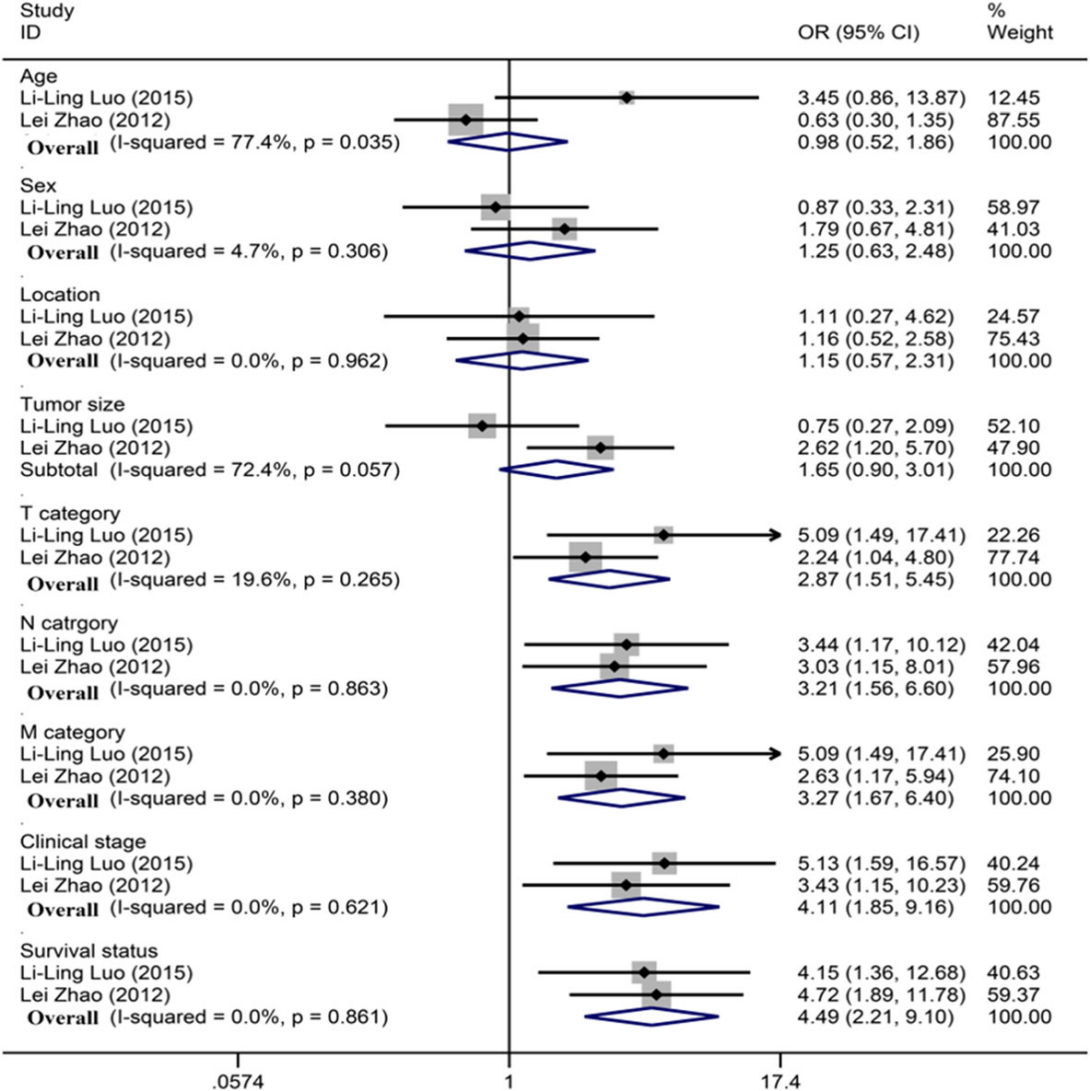
Forest plot of study estimating the association between the low IGFBP-3 level and clinic pathological characteristics of esophageal cancer

With respect to clinical pathological characteristics, the results were displayed in Table 2. Although the ORs for age, gender, location, and tumor size (Fig. 4, age: OR = 0.980, 95% CI: 0.520–1.860; gender: OR = 1.250, 95% CI: 0.630–2.480; location: OR = 1.145, 95% CI: 0.568–2.307; tumor size: OR = 1.650, 95% CI: 0.900–3.010) were higher than 1, all their corresponding *p* were higher than 0.05, which demonstrated that for esophageal cancer patients, no significant association was detected between the low IGFBP-3 level and the age, gender, tumor location, tumor size of patients. For survival status, the OR was 4.490 (95% CI: 2.207–9.093, *p* = 0.000, Fig. 4), signifying that the low IGFBP-3 level was correlated with the survival status of patients, and esophageal cancer patients with low IGFBP-3 level occupied a lower survival rate.

In terms of the cancer category, we applied the fixed-effects model for the calculation of OR and 95% CI due to the low heterogeneity, and the results were shown in Table 2. For patients in *T* category, the OR was 2.870 (95% CI: 1.510–5.450, *p* = 0.005, Fig. 4), implying that for esophageal cancer patients in *T* category, patients whose tumors extended invasion in other organs had lower IGFBP-3 level than those without invasion in other organs. For patients in *N* category, the OR was 3.211 (95% CI: 1.561–6.607, *p* = 0.002, Fig. 4), indicating that the IGFBP-3 level in patients of *N* category whose tumor cells had lymph node metastasis were significantly lower than that in patients without lymph nodes in tumor cells. For patients in *M* category, the OR was 3.270 (95% CI: 1.670–6.400, *p* = 0.001, Fig. 4), suggesting that for esophageal cancer patients in *M* category, patients whose tumor cells metastasized to distant organs (beyond regional lymph nodes) had lower IGFBP-3 level than those without distant metastases. For patients in TNM category, the OR was 4.110 (95% CI: 1.850–9.160, *p* = 0.000, Fig. 4), signifying that for esophageal cancer patients in TNM category, the IGFBP-3 level in patients with III–IV tumor stage was significantly lower than that in patients with other tumor stages.

## Publication bias

**Fig. 5:**
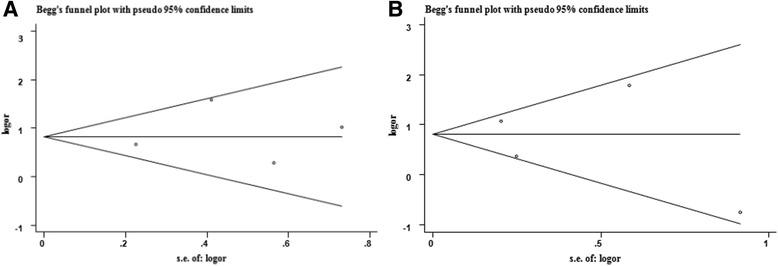
Funnel plots for study investigating the association between the low IGFBP-3 level and cancer risk (**a**) and 3-year survival rate (**b**)

Both Begg’s test and Egger’s test were used to examine the publication bias, the results were exhibited in Table 2. For cancer risk and 3-year survival rate, results from both Begg’s test and Egger’s test indicated no significant publication bias, and the corresponding funnel plots were shown in Fig. 5a, b. With respect to other parameters, it was unnecessary to construct the funnel plots due to the small number of pooled studies, yet all the corresponding *p* were higher than 0.05, inferring that there was no significant publication bias.

## Discussion

In order to comprehensively assess the correlation between low IGFBP-3 level and the risk, overall survival and clinical pathological characteristics of esophageal cancer, we conducted the current meta-analysis incorporating seven eligible studies. Our results show that for the risk of cancer, individuals with low IGFBP-3 level are more likely to suffer from esophageal cancer. As for the overall survival, the esophageal cancer patients in low IGFBP-3 level have lower 3-year survival rate than those in relatively high IGFBP-3 level. Regarding to clinical pathological characteristics, for *T* category, patients whose tumors have extended invasion in other organs are more likely to have low IGFBP-3 level; for *N* category, patients whose tumor cells have regional lymph nodes are more likely to have low IGFBP-3 level; for M category, patients whose tumor cells metastasize to distant organs are more likely to have low IGFBP-3 level; for TNM category, patients in III–IVstage are more likely to have low IGFBP-3 level; for survival status, patients in low IGFBP-3 level are more likely to be in the dead survival status; whereas the low IGFBP-3 level is not associated with the age, gender, tumor location, and size of esophageal cancer patients.

Esophageal cancer, an aggressive carcinoma, is the eighth most common cancer in the world [24]. The lack of serosa in the esophageal wall leads to no anatomical barrier for the spread and aggressiveness of esophageal cancer, which contributes to the rapid extension into the adjacent structures such as the neck, larynx, aorta, thyroid gland, lung, and so on [25]. Esophageal cancer can spread by not only directly extending but also lymphatic spread and hematogenous metastasis [25]. The management of esophageal cancer largely depends on the preoperative assessment, and the standardized techniques of assessment include upper endoscopy, high-resolution contrast computed tomography (CT) scan, 18 fluoro-2-deoxyglucose positron emission tomography (FDG-PET) scan, and endoscopic ultrasonography (EUS) [26].

IGFBPs include IGF high-affinity-binding proteins (IGFBP1-6) and IGF low-affinity IGFBP-related proteins (IGFBP-rP1-10) [27], which prolong the half-life of IGF1 in circulation, and regulate the access of IGF1 to its receptor by carrying circulating IGF1 peptide and interacting with other proteins such as matrix metalloproteinases (MMPs) [28]. IGFBP-3, the most abundant circulating IGFBP, is a critical regulatory molecule of IGF system [12, 29]. The abnormal expression or dysfunction have been associated with a verity of tumors [29]. Through both IGF-dependent and IGF-independent pathways, IGFBP-3 possesses anti-proliferative, pro-apoptotic functions [30]. It was suggested that Smad3-P27/P21-cyclin E1/CDK2-phosphorylated retinoblastoma protein pathways might be involved in IGFBP-3-mediated radiosensitivity transition in esophageal squamous cell carcinoma (ESCC) the most prevalent histologic type of esophageal cancer in China and eastern countries. A study of 500 ESCC patients indicated that IGFBP-3 SNP rs2270628 may be associated with ESCC in the Chinese Han population [31]. The levels of IGFBP-3 protein expression in ESCC tissues were decreased [32]. In addition, Yilmaz et al. performed a case-control study and observed that the level of IGFBP-3 was related with esophageal cancer risk and patients with esophageal cancer had significantly lower IGFBP-3 levels, compared with the control group (healthy people) [21]. Our study also revealed that the IGFBP-3 level in patients with esophageal cancer was significantly lower than that in healthy participants.

Overall survival (OS), an easily appreciated parameter, remains the strongest trial endpoint in cancer clinical trials, which is independent of bias resulting from the definition of progression, approaches of evaluation, and clinical assessment [33]. Previous studies implied that for esophageal cancer patients, the elevated IGFPB-3 level was significantly associated with improved overall survival [15, 17], which was inconsistent with the related study published in 2004 [16]. However, with large sample size by pooling relevant data together, our meta-analysis indicated that low IGFPB-3 level was predictive for poor overall survival for esophageal cancer patients. Furthermore, significant association was observed between the low IGFBP-3 level and clinical pathological characteristics including the tumor category and survival status rather than the age, gender, tumor location, and tumor size of patients in current meta-analysis.

Serum human relaxin 2, which impacts on the remodeling of several tissue components including matrix metalloproteinase, extracellular matrix, and collagen, has been discovered as a potential prognostic factor for esophageal cancer, and patients with higher human relaxin 2 level had shorter survival period, more distant metastasis and higher tumor stage [25]. A previous meta-analysis demonstrated that the high level of serum C-reactive protein were significantly correlated with poor overall survival in esophageal cancer patients [34]. The identification of matrix metalloproteinase-7 protein was also proved as a promising biomarker for esophageal cancer progression [35]. Our study revealed that the IGFBP-3 was another potential diagnostic and prognostic biomarker for esophageal cancer.

The present study is the first meta-analysis to assess the association between the low IGFBP-3 level and the risk, overall survival, and clinical pathological characteristics of esophageal cancer. However, some limitations exist in the current study. Firstly, the test methods of IGFBP-3 level were not exactly the same in the six included studies, and with more relevant studies becoming available, subgroup analysis stratified by different detection methods of IGFBP-3 level should be made for a more reliable estimation. Secondly, the cut-off value of IGFBP-3 level for the determination of esophageal cancer was not quite the same, which might result in some bias for our meta-analysis. Additionally, the unpublished articles were not retrieved in our study.

## Conclusion

The current meta-analysis suggests that the lower IGFBP-3 level is significantly associated with higher cancer risk, lower 3-year survival rate, more advanced tumor stages, and more distant metastasis. The low IGFBP-3 level is a promising predictor for high-risk, poor prognosis, and unfavorable tumor stage and metastasis of esophageal cancer.

## Abbreviations

CI: Confidence interval
CT: Computed tomography
ELISA: Enzyme linked immunosorbent assay
EUS: Endoscopic ultrasonography
FCM: Flow cytometry
FDG-PET: 18 fluoro-2-deoxyglucose positron emission tomography
IGF: Insulin-like growth factor
IGFBPs: Insulin-like growth factor binding proteins
IHC: Immunohistochemical staining
MeSH: Medical subject headings
OR: Odds ratios
OS: Overall survival
RR: Relative risk
RT-PCR: Reverse transcription-polymerase chain reaction
